# A Framework for Implementing the National Diabetes Prevention Program in Los Angeles County

**DOI:** 10.5888/pcd14.160433

**Published:** 2017-08-24

**Authors:** Jennifer T. Mosst, Amelia DeFosset, Lauren Gase, Laura Baetscher, Tony Kuo

**Affiliations:** 1 Division of Chronic Disease and Injury Prevention, Los Angeles County Department of Public Health, Los Angeles, California; 2 Department of Epidemiology, UCLA Fielding School of Public Health, Los Angeles, California; 3 Department of Family Medicine, David Geffen School of Medicine at UCLA, Los Angeles, California

## Abstract

**Introduction:**

Preventing type 2 diabetes is a public health priority in the United States. An estimated 86 million Americans aged 20 years or older have prediabetes, 90% of whom are unaware they have it. The National Diabetes Prevention Program (NDPP) has the potential to reduce the incidence of type 2 diabetes; however, little is known about the best way to institutionalize such a program in a jurisdiction with a racially/ethnically diverse population. The objective of this study was to develop a practice-grounded framework for implementing the NDPP in Los Angeles County.

**Methods:**

In 2015, the Los Angeles County Department of Public Health (LACDPH) partnered with Ad Lucem Consulting to conduct a 3-stage formative assessment that consisted of 1) in-depth interviews with key informants representing community-based organizations to learn about their experiences implementing the NDPP and similar lifestyle-change programs and 2) 2 strategic planning sessions to obtain input and feedback from the Los Angeles County Diabetes Prevention Coalition. LACDPH identified core activities to increase identification of people with type 2 diabetes and referral and enrollment of eligible populations in the NDPP.

**Results:**

We worked with LACDPH and key informants to develop a 3-pronged framework of core activities to implement NDPP: expanding outreach and education, improving health care referral systems and protocols, and increasing access to and insurance coverage for NDPP. The framework will use a diverse partner network to advance these strategies.

**Conclusion:**

The framework has the potential to identify people with prediabetes and to expand NDPP among priority populations in Los Angeles County and other large jurisdictions by using a diverse partner network

## Introduction

Preventing type 2 diabetes is a public health priority in the United States (1). Prediabetes occurs when a person’s blood glucose level is higher than normal (fasting blood glucose level of 100–125 mg/dL [5.6 to 7.0 mmol/L]), putting the person at increased risk for heart disease, stroke, and developing type 2 diabetes (2,3). An estimated 86 million Americans aged 20 years or older have prediabetes, and 90% of those do not know they have it (3). In 2012, diabetes and prediabetes were estimated to cost $245 billion nationally and $32.3 billion in California, through direct medical spending and lost productivity (4).

Prediabetes can be reversed through lifestyle modifications (3,4). For example, the National Diabetes Prevention Program (NDPP), an intensive lifestyle-change program focused on improving diet and physical activity, can delay the onset of diabetes among those at risk (5,6). In response, the Centers for Disease Control and Prevention (CDC) made significant investments in state and local efforts to translate NDPP into community settings and to grow the program in the Los Angeles County (7). In 2014, the Los Angeles County Department of Public Health (LACDPH) was selected as a large-city participant in CDC’s cooperative agreement 1422, State and Local Public Health Actions to Prevent Obesity, Diabetes, and Heart Disease and Stroke. In Los Angeles this program is called the Chronic Disease Prevention Strategy. One of the program’s primary aims is to expand access to and participation in NDPP in Los Angeles. To advance this goal, LACDPH partnered with the YMCA of Metropolitan Los Angeles to co-lead the Los Angeles County Diabetes Prevention Coalition (LACDPC), which was established in 2012 to help the YMCA and others expand enrollment in the NDPP.

Although LACDPC could serve as a powerful vehicle in Los Angeles to implement NDPP, the actions the coalition should take to institutionalize the program across this large, racially/ethnically diverse jurisdiction were unclear. In particular, little is known about the best ways to implement such a program among high-risk, high-burden priority populations (ie, those who have prediabetes or uncontrolled high blood pressure, those who live in low-income communities, and those in racial/ethnic minority populations who experience disparities in access to and quality of care) (7). To develop a practice-grounded framework for the Los Angeles County NDPP, we conducted a 3-stage formative assessment. This assessment sought to answer the following questions: 1) what core activities are needed to identify, refer, and enroll eligible participants in the NDPP, including establishing payment options to offset costs?, and 2) what key partners are needed to advance this work?

## Methods

In summer 2015, LACDPH partnered with Ad Lucem Consulting (ALC) to conduct a 3-phase formative assessment to inform objectives for the Chronic Disease Prevention Strategy, including identifying a strategic plan for the LACDPC. The assessment team, which included staff members from LACDPH and ALC, used an outcomes-focused approach (8), first defining the desired goal — increasing identification, referral, and enrollment of eligible populations into the NDPP — and then working backward to identify key activities and partners. In phase 1, ALC conducted in-depth interviews with key informants to learn about their experiences implementing NDPP and similar lifestyle-change programs. In phase 2, ALC and LACDPH presented results from these interviews to LACDPC for input and feedback. In phase 3, LACDPH synthesized results from the interviews and coalition dialogues into a practice-based framework. The project was reviewed and considered exempt by the LACDPH internal review board.

### Phase 1: Qualitative key informant interviews

Eligible participants were recruited through a multistage sampling process that combined snowball and maximum variation techniques (9). The goal was to identify leaders in prevention and management in the United States working in various roles (ie, practitioners, policy makers, researchers, and funders) and organizations (eg, health care agencies, health plans, health departments, community-based organizations [CBOs]). First, LACDPH, along with contacts at the American Diabetes Association, American Association of Diabetes Educators, and CDC, provided ALC with a list of potential participants who were individuals or organizations that had field experience implementing the NDPP or other chronic disease prevention or management programs, or who had conducted NDPP-focused research and evaluation. An initial round of interviews was carried out with those listed: 1) organizations that were CDC-recognized NDPP providers (if applicable), and 2) individuals or organizations that had experience serving priority populations. Interviewees in the initial round were then asked to identify other individuals or organizations meeting the inclusion criteria. Recruitment continued until the sample included a balance of participants in terms of roles and organizational types. ALC recruited all potential interviewees through email, with follow-up telephone calls, as needed.

Of the 45 experts identified, 33 consented to participate. ALC conducted interviews with representatives from health departments and government agencies (9 respondents), health care providers and health plans (9 respondents), nonprofit and CBOs (8 respondents), academic institutions (4 respondents), and funders (3 respondents). Most participants were from Los Angeles (18 respondents), and the others (15 respondents) were from large metropolitan cities (populations of 8 million or more) or large states (populations of more than 19 million).

Four trained ALC interviewers conducted all interviews by telephone in August and September 2015. The interview guide included 7 primary open-ended questions and associated probes focused on understanding 1) how to increase referrals to NDPP, 2) how to increase enrollment in NDPP, 3) models for NDPP delivery, 4) barriers to implementing NDPP, 5) models for reimbursement and coverage of NDPP, 6) expanding the pool of NDPP providers, and 7) the role of health departments in implementing NDPP. Each interview was conducted by one interviewer and lasted approximately one hour, during which time the interviewer typed notes (transcripts) to record responses verbatim. The transcripts were then uploaded into Atlas.ti (Scientific Software Development, GmbH) for qualitative analysis. First, 2 interviewers developed a list of thematic codes based on the interview questions. Second, the 4 interviewers independently coded transcripts in batches, meeting 4 times as a full group to reconcile coding, refine the coding scheme, and group codes into themes in 4 predefined areas relevant to developing a strategic plan for LACDPC 1): ways to increase demand for the NDPP among patients and providers, 2), ways to engage the health care system, 3) ways to increase the supply and capacity of NDPP providers, and 4) ways to conduct NDPP implementation research and evaluation.

### Phase 2: Input from the Los Angeles County Diabetes Prevention Coalition

ALC and LACDPH presented the themes developed from the interviews during phase 1 to LACDPC during 2 in-person strategic planning sessions in December 2015 and February 2016. Sixteen coalition members, representing 11 institutions, participated. The sessions, which lasted approximately 3 hours each, focused on systematically generating input on key activities that were most important and relevant to advancing diabetes prevention in Los Angeles. We solicited reactions from coalition members with questions such as, Is this activity important?, What partners are needed to implement these activities?, What else could complement these activities?, and How can these activities be applied to diabetes prevention work locally in Los Angeles?. During each session, one trained note taker recorded participants’ discussion. ALC and LACDPH staff members then met to review and summarize the notes, which were then shared via email with the full coalition for final input and confirmation.

### Phase 3: Framework development

Two LACDPH team members conducted a holistic analysis of the data generated during phases 1 and 2 and synthesized these into a framework that identified the core activities needed to increase identification, referral, and enrollment of eligible populations into the NDPP in Los Angeles. The 2 team members were guided by, but not bound to, analyses conducted by ALC in phase 1 to identify the themes that were presented to the coalition. LACDPH staff compiled interview transcripts and notes from coalition sessions into a master document. Two team members reviewed this document independently, working inductively to assign descriptive codes to segments of text (10,11). After independent review, the 2 team members met to discuss codes, reconcile differences in interpretation, and develop a core set of activities. The team prioritized activities that were identified by both multiple interview participants and members of LACDPC. The team grouped the activities into overarching domains and developed a visual representation to show how they relate to one another and the desired outcomes.

## Results

Key informant interviews generated themes in phase 1, and the coalition generated additional input in phase 2 (Table). The framework developed in phase 3 consists of 3 domains 1): expanding outreach and education, 2) improving health care referral systems and protocols, and 3) increasing access to and insurance coverage for NDPP. The framework relies on a diverse partner network for advancing these strategies (Figure).

**Table:** Recommended Actions for Implementing the National Diabetes Prevention Program (NDPP): Results From Key Informant (N = 33) Interviews and Discussions With the Los Angeles County Diabetes Prevention Coalition^a^

Phase 1. Activities Identified from Key Informant Interviews (N = 33)^a^	Phase 2. Feedback from the LAC Diabetes Prevention Coalition
**Area 1: Ways to Increase Demand for the NDPP Framework Domain: Expand Outreach and Education**	
**Increase prediabetes awareness**	
•Promote the Ad Council American Diabetes Association (ADA)/American Medical Association (AMA)/Centers for Disease Control and Prevention (CDC) National Prediabetes Awareness Campaign to increase knowledge and awareness of the NDPP (n = 9). •Work with regional NDPP provider organizations to encourage the use of traditional and social media channels to distribute the campaign (n = 17). •Partner with regional media outlets reaching high-risk ethnic populations to promote the campaign (n = 10).	•Develop a strategy for the coalition to promote the campaign in the region. •Develop relationships with local media outlets to promote the campaign. •Work to tailor the campaign to have more of a local focus and message.
**Engage trusted, culturally relevant organizations and individuals to promote prediabetes screening and the NDPP**	
•Adopt and disseminate non-invasive risk assessments screeners for prediabetes (eg, ADA and CDC prediabetes risk screeners) (n = 33). •Enlist organizations and individuals (eg, *promotoras*, diabetes educators, churches, community groups, health care systems) to conduct prediabetes screenings and concurrent NDPP promotion and referral (n = 25).	•Work with local health department to disseminate resources for identifying patient risk of prediabetes. •Host regional training on identifying prediabetes risk with community health workers, *promotoras*, and health navigators. •Partner with local community clinic organizations and community-based organizations (CBOs) to provide educational resources and training to increase screening and referrals. •Partner with Covered California to conduct screening of prediabetes risk with individuals applying for health insurance.
**Outreach to employers to promote NDPP**	
•Identify and develop resources for how to work with local employers to implement the NDPP (n = 20). •Work with employers to identify opportunities to offer onsite classes for employees and/or refer employees to NDPP programs in the region (n = 20). •Develop materials and resources on return on investment (ROI) of the NDPP, including impacts on absenteeism and worker productivity (n = 21). •Partner with regional organizations that work directly with large employers (eg, Los Angeles Chamber of Commerce Health Committee chapters, unions) (n = 8). •Facilitate healthy competition, for example, invite employers to publicize NDPP success stories (n = 6).	•Work with local health department to identify organizations (ie, employers, nonprofits) to adopt the NDPP for employees. •Partner with third-party groups to identify organizations interested in scaling the NDPP. •Host a convening of regional employer human resource departments to educate them about the NDPP and identify opportunities to implement the program. •Partner with local health plans to identify employers with robust worksite wellness options to discuss providing NDPP services on site for employees. •Identify models for how NDPP providers can work with employers in the region.
**Area 2: Ways to Engage Health Care Systems Framework Domain: Improve Health Care Referrals Systems and Protocols**	
**Educate health care providers on prediabetes screening and the NDPP**	
•Survey clinics to understand local health system approaches to identifying, referring, and enrolling individuals into the NDPP (n = 18). •Identify key individuals/organizations to facilitate conversations with health care systems (eg, chief medical officers, Community Clinic Associations) to identify education needs (n = 20). •Develop (as needed) and disseminate materials and toolkits for educating providers on the NDPP (eg, AMA CDC Prevent Diabetes STAT Toolkit, US Preventive Services Taskforce prediabetes screening guidelines) (n = 30). •Develop continuing medical education resources around prediabetes risk and identification and referral to the NDPP (n = 30).	•Work with local health department to develop/adapt materials and provide training and technical assistance for educating providers on the NDPP. •Partner with local health department to develop continuing medical education for providers and lay practitioners on the NDPP. •Partner with local health department to develop and pilot test NDPPs within local health systems.
**Promote use of electronic medical records (EMRs) to generate lists of patients with prediabetes and generate automatic referrals**	
•Develop mechanisms and protocol for local health care systems to: 1) query EMRs to generate lists of patients with prediabetes (n = 20); use EMRs to generate patient referrals to the NDPP and other programs (n = 13); 3) create feedback loops between NDPP providers and health care providers to track patient NDPP enrollment and progress (n = 25). •Partner with organizations implementing EMRs (eg, CBOs, NDPP providers) to develop infrastructure for identification, referral, and enrollment in NDPP (n = 14).	•Partner with local health departments to develop/adapt materials for health systems to use to educate providers on the NDPP. •Partner with local health care systems to develop infrastructure to screen, refer, and enroll patients into the NDPP. •Develop a comprehensive database for chronic disease prevention and management programs in the region for providers to use to refer patients. •Partner with meaningful use governing boards to create practice-based models for screening and referring into the NDPP. •Partner with local health care systems to evaluate and identify best practices for implementing NDPP in the region.
**Create financial and quality measure incentives for addressing prediabetes (n = 33)**	
•Partner with health and medical groups (eg, CDC, Community Clinic Association of Los Angeles County) to promote: 1) including prediabetes screening in National Committee for Quality Assurance regulatory requirements for quality of care; 2) creating Health Care Effectiveness Data and Information Set measures for NDPP components; 4) incorporating NDPP in patient-centered medical home certification. •Partner with research institutions to conduct an economic analysis of the NDPP looking at ROI. •Engage health care systems and providers in quality improvement projects that focus on NDPP referral processes.	•Develop a white paper looking at the ROI for health care system implementing the NDPP. •Develop a white paper on facilitators and barriers to implementing the NDPP in health care settings.
**Area 3: Ways to Increase the Supply and Capacity of NDPP Providers Framework Domain: Increase Access to and Insurance Coverage for the NDPP**	
**Expand the network of CDC-recognized NDPP providers**	
•Develop resource inventory to include maps of current NDPPs in the region (n = 10). •Identify community organizations in high-need areas who may be interested in developing programs like the NDPP (n = 7). •Develop resources and training opportunities on the CDC NDPP recognition process to make it understandable and accessible to local community organizations (n = 20). •Identify funding sources to provide lifestyle coach training with no costs to participants (n = 23). •Work with small regional organizations serving low-income and ethnic populations to become recognized NDPP providers (n = 7). •Conduct training and technical assistance with regional organizations to obtain CDC recognition (n = 4). •Convene local CBOs and other potential provider organizations to discuss barriers and facilitators to implementing the NDPP (n = 17). •Work with existing recognized NDPP programs to: 1) expand their NDPPs to hard-to-reach areas; 2) develop a train-the-trainer model for NDPP program development and recognition; 3) partner with regional CBOs to host NDPPs for community members. •Identify funding opportunities to expand NDPP efforts (eg, ADA, AMA, regional hospital community benefits departments) (n = 33).	•Create resource inventory to identify and map NDPP providers in the region. •Facilitate resources and/or funding for CBOs to become recognized NDPP providers. •Provide trainings and technical assistance on how organizations can become CDC-recognized, especially with regard to data collection and reporting. •Identify and reach out to organizations working on chronic disease management to see if they are interested in providing NDPP. •Develop budget templates that organizations can use when establishing NDPPs. •Develop best practices resources of what has worked with providers regionally and locally in developing NDPP efforts. •Work with members of the coalition to identify funding opportunities (hospital benefits departments, etc.) for developing NDPPs.
**Improve the cultural relevance of NDPP**	
•Tailor NDPP curricula/materials to meet the needs of a variety of cultural and linguistic groups (n = 27). •Identify top 5 languages/cultures in the region and translate NDPP materials into those priority languages (n = 27). •Train lifestyle coaches to provide curriculum in priority languages identified for the region (n = 10). •Identify culturally competent lifestyle coaches to provide NDPP in priority languages (n = 10).	•Facilitate resources for CBOs to adapt/create materials and toolkits for NDPP implementation. •Adapt NDPP lifestyle coach training opportunities to include training options in languages identified as priority in the region. •Provide training for NDPP providers to include other issues impacting participants (eg, mental health).
**Evaluate local prediabetes data**	
•Identify methods for collecting prediabetes prevalence data (n = 8). •Monitor ongoing data collection and analysis of regional NDPP providers (n = 8). •Report and disseminate prediabetes data to help identify best practices (n = 3). •Publish papers on facilitators and barriers to implementing the NDPP (n = 10).	•Develop a survey for key stakeholders to understand the local impacts of prediabetes and the need for the NDPP.
**Conduct NDPP implementation evaluation in existing and new pilot sites**	
•Develop an evaluation toolkit that can be implemented in across NDPP sites that includes information on what data to collect, data sources, how to analyze data, and how to report to CDC (n = 13). •Develop resources to measure: 1) enrollment and retention: Individuals’ decision-making processes; 2) program delivery (providers, cost, location, languages, frequency, use of personal scales); 3) impact (adoption of healthy behaviors, progression to diabetes). •Evaluate fidelity of NDPP implementation among local NDPP providers (n = 18).	•Convene key stakeholders to identify and prioritize data sources for evaluating implementation of the NDPP in the region. •Develop platform to facilitate sharing of NDPP program data. •Develop an evaluation plan for NDPP providers to use to measure success. •Conduct research of NDPP implementation efforts and disseminate reports on findings. •Disseminate finding of outcome data of existing pilot programs. •Develop and disseminate best practices from data collected through pilot projects in the region.

Abbreviations: LAC, Los Angeles County; NDPP, National Diabetes Prevention Program; STAT, Screen/Test/Act Today; CBOs, community-based organizations.

a All themes and actions were generated from response rates from participants in key informant interviews and the in-person strategic planning session. Numbers in parentheses indicate how often the recommended action was recorded during the key informant interviews.

**Figure:**
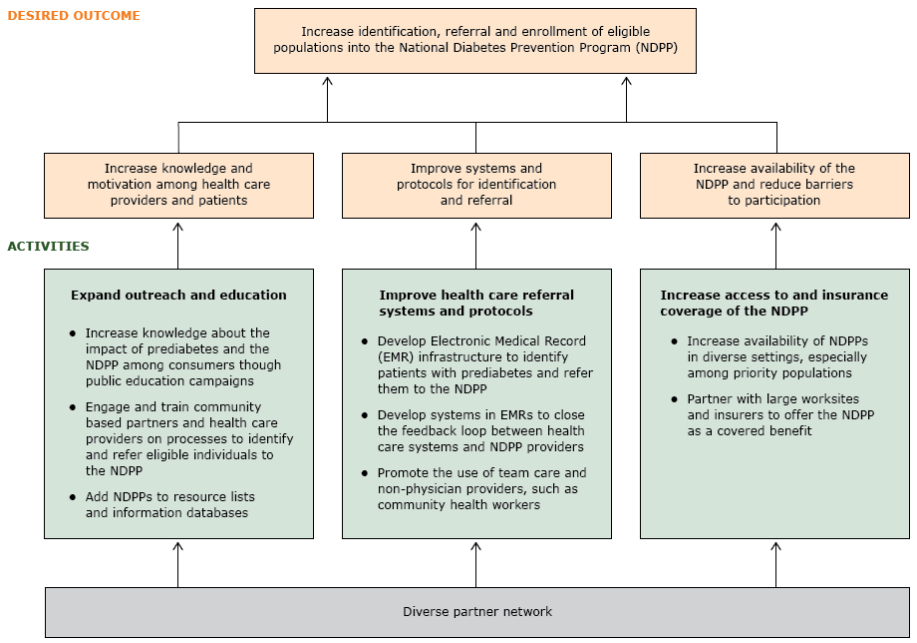
Framework for implementing the National Diabetes Prevention Program (NDPP) in Los Angeles County.

**Expand outreach and education.** The first domain emphasizes the importance of providing education and training to the public and community-based partners and health care providers to increase knowledge and awareness of prediabetes and of the NDPP. LACDPH identified 3 activities to expand outreach and education. First, to help increase knowledge among the general public, public education campaigns such as the Ad Council’s Diabetes Prevention Campaign (www.adcouncil.org/Our-Campaigns/Health/Type-2-Diabetes-Prevention) are needed to emphasize the importance of preventing diabetes and the value of lifestyle-change programs. Participants recommended creating and disseminating a national message that includes an appropriate local-level focus to help empower those most affected by prediabetes to talk to their health care providers (ie, helping to increase demand for the NDPP). Second, participants recommended developing educational resources targeting community partners and health care providers (ie, physicians, community health workers, *promotoras*, health navigators, and large employers). Participants emphasized the importance of training community-based partners and health care providers on the content and scope of NDPP, on tools to screen people for prediabetes, and on integrating screening and referral tools into practice. Existing training resources, such as the American Medical Association–CDC Prevent Diabetes STAT toolkit (https://preventdiabetesstat.org/toolkit.html), were identified as important tools for increasing knowledge and enhancing the referral process. Third, participants recommended creating an NDPP resource inventory (ie, informational resource lists and databases) as a complementary activity to increase knowledge and awareness of programs, including where, when, and in what languages classes are offered.

**Improve health care referral systems and protocols**. The second domain emphasizes the need to create referral systems and protocols for health care providers to refer and identify at-risk patients to NDPP. LACDPH identified 3 activities to improve health care referral systems and protocols. First, participants recommended enhancing the existing electronic medical record (EMR) system to identify patients with prediabetes and refer those eligible to local health care providers participating in NDPP. Participants recommended developing mechanisms to conduct regular queries of EMRs to identify patients at risk for prediabetes and link them to NDPP providers through an automatic referral process. CBOs could use similar electronic processes to screen people for diabetes risk and refer those eligible directly to local NDPP providers. Second, participants recommended modifying EMRs to create feedback loops between the health system and local NDPP providers. These feedback loops would help enhance bidirectional communication between health systems and community-based NDPP providers to more effectively manage patient care. Third, participants recommended expanding the use of team care and nonphysician providers, especially community health workers, to identify and refer patients to NDPP. Participants emphasized the relevance of using team-based approaches to reduce provider burden and enhance coordination of care for patients to improve processes for identifying and referring patients to NDPP.

**Increase access to and insurance coverage of the NDPP.** The third domain emphasizes the importance of increasing access to NDPP and insurance coverage for health care associated with participation in NDPP. Participants described the lack of program options (eg, delivery formats, language options) and insurance coverage for NDPP as significant barriers to enrollment, especially for priority populations, including those in low-income communities and those who speak languages other than English. LACDPH identified 2 activities to increase access and coverage. First, participants recommended increasing the availability of NDPP providers in diverse settings and expanding the network of NDPP providers by 1) providing technical assistance to new organizations to administer the NDPP and 2) helping current providers increase their reach in priority areas. Participants identified the need to improve the cultural relevance of NDPP, including training lifestyle coaches that represent the cultures and languages of high-risk populations and developing and disseminating a culturally diverse resource guide for participants to augment NDPP and support the adoption of healthy behaviors. Recommendations were made to offer the program in identified priority languages: Spanish, Chinese, and Korean. Second, participants described the need to partner with large worksites and insurers to offer NDPP as a covered insurance benefit. Working directly with employers can help facilitate access to NDPP and insurance coverage for NDPP health care services. Interview participants felt that employer-based NDPP programs (ie, offering NDPP directly at targeted worksites) were a convenient way to engage potential program participants, implement screening protocols, and facilitate coverage of the program. Insurance providers were identified as another key partner in helping to remove cost barriers to participation in the NDPP. The need to conduct additional research and evaluation to identify NDPP models that meet the need of payers by demonstrating return on investment is a high priority. In addition, creating financial and quality incentives, such as a Healthcare Effectiveness Data and Information Set measure for prediabetes, might facilitate increased access to and insurance coverage of NDPP-related health care.

**Partner network.** The framework relies on a diverse partner network to implement NDPP. Participants described diverse partnerships to facilitate capacity building among providers, assist with the development of educational resources for training, increase awareness of NDPP, and provide resources for increasing access to the program. Participants stressed the importance of partnerships among health care organizations, local and national government entities, nonprofit organizations, CBOs, payers, local funding organizations, and NDPP provider organizations. Additionally, participants emphasized the need to work with local NDPP providers to pilot programs and test payment models to build the case for insurance coverage. Participants from LACDPC recognized the importance of their role in facilitating many of these partnerships by convening key stakeholders (ie, NDPP providers, insurers, academic partners, health care providers, government, CBOs) and working to grow the coalition to increase the diversity of organizations and member expertise.

## Discussion

We described a 3-pronged framework to increase the identification, referral, and enrollment of participants in NDPP: expanding outreach and education, improving health care referral systems and protocols, and increasing access to and insurance coverage of the NDPP. The framework relies on a diverse partner network in advancing this work. The framework provides a roadmap for the work of LACDPH and LACDPC.

Increasing uptake of the NDPP in Los Angeles will require the use of a multipronged approach that simultaneously focuses on increasing availability of and demand for the program while reducing potential barriers to program participation. Such an approach echoes calls to action from leaders in community translation and in clinical prevention; these calls have separately included recommended actions to increase awareness among patients and health care providers about the risk of prediabetes (12), to enhance clinical systems to institutionalize the novel prevention approach (13,14), or to implement varied and sustainable program and payment models to ensure that the NDPP is available and accessible to the full population in need (12,15). The framework developed in this study synthesized key informant recommendations into a single practice-based model that emphasizes the importance of advancing the 3 prongs of the framework concurrently so that they are mutually reinforcing.

Our study suggests that diverse partners are needed to implement the framework. Best-practice recommendations to implement evidence-based programs reinforce the importance of early and meaningful involvement from a full range of stakeholders (8); our study suggests that key stakeholders in the implementation of NDPP should include representatives from business, health systems, NDPP providers, government, community, education/academia, and philanthropy. A coordinated, collaborative effort that includes these groups (the foundations of this effort were developed locally by LACDPC [16]) will be needed to advance the multifaceted and mutually reinforcing strategies necessary to implement NDPP in Los Angeles County. We anticipate that LACDPC can build on this study’s framework with input from community members to address the complex health problem that diabetes poses (16–23).

The formative assessment process and resulting framework described in this study has been useful in Los Angeles for organizing and developing plans to implement NDPP (21). The framework is currently being implemented by LACDPC, which has adopted a subcommittee structure to advance activities in each of the framework’s 3 domains. Other evidence-based health promotion programs have suggested the need for additional actions, such as assessing local conditions and capacity (24,25). However, this type of planning action did not emerge as a priority in our study. One potential reason for this is that the multistage process of vetting broader national perspectives (collected in phase 1) with local stakeholders (through the planning sessions held in phase 2), resulted in a framework that reflects existing conditions in Los Angeles County and steps needed to implement the framework. Although more work is needed to systematically examine the framework’s local usefulness and impact, other interested jurisdictions may wish to adapt the formative assessment process used in this study to develop practice-based frameworks that reflect their own local needs.

This study has several limitations. First, although the strategic planning process offered an opportunity to confirm and enrich interview data, the scope of the information presented during these sessions was limited by the initial interview guide. A more open-ended process could have acquired more information. Similarly, soliciting the perspective of potential NDPP participants could have provided information on barriers and facilitators to program enrollment; however, because our study focus was to identify key actions organizations could take expand NDPP, collecting such data from potential participants was beyond the scope of this assessment. Second, the assessment was guided by an outcomes-focused, practice-grounded approach (7), which sought to identify concrete action steps to increase the identification, referral, and enrollment of eligible populations into NDPP in Los Angeles. A theoretical model was not used to guide the assessment. Finally, although key informant interviews were conducted with various local and national experts, viewpoints from Los Angeles were heavily represented in the development process. Additional efforts are needed to determine whether our framework can be useful for other jurisdictions.

A comprehensive framework that identifies the core activities and partners needed to implement the NDPP regionally can provide a useful platform to organize collaborative efforts. Other jurisdictions can use the processes and results in this study to help advance evidence-based, lifestyle-change programs such as the NDPP in their communities.
